# How Language and Human Altruism Evolved Hand in Hand — The Backchannel Hypothesis

**DOI:** 10.3389/fpsyg.2022.735375

**Published:** 2022-02-10

**Authors:** Till Nikolaus von Heiseler

**Affiliations:** Department of Philosophy, Institute of Philosophy, Humboldt-Universität zu Berlin, Berlin, Germany

**Keywords:** virtue signaling, reputation, cooperation, backchannel hypothesis, altruism, equilibrium selection, language evolution, storytelling

## Abstract

This paper contributes to two debates: the debate about language evolution and the debate about the foundations of human collaboration. While both cooperation and language may give the impression of being adaptations that evolved for the “good of the group,” it is well established that the evolution of complex traits cannot be a *direct* result of group selection. In this paper I suggest how this tension can be solved: both language and cooperation evolved in a unique two-level evolutionary system which was triggered by a well-documented geological event—the drying out of the climate—in East Africa, which subsequently reduced the intermating between groups and thus made it possible that the mechanism that produced differences between groups (including social forms of selection such as female choice) could be the target of natural selection on the group level. If a social form of selection (e.g., sexual selection) produced differences in fitness between groups, the displacement process between groups would indirectly select those forms of social selection that produce groups that would displace all others. The main hypothesis presented in this paper is that, in this situation, a backchannel between the two levels of selection naturally evolves. A backchannel between the two levels would, for example, emerge when sexual selection (or any other form of social selection) was sensitive to the individual’s contribution to the group. Examples of systems utilizing a backchannel are nerve cells being better nourished when used more frequently, enabling them to be conducive to the survival of the whole organism, or a law firm in which all employees get paid to the extent that they contribute to the survival and success of the firm. In both cases, the selection on the higher level informs the selection on the lower level. The aim of the paper is to illuminate these rather opaque claims, to which the reader probably has many objections in this abridged form.

## Introduction

### What Needs to Be Explained

Cooperation and language have three things in common: (1) They are considered to be essential for being human, (2) their evolution is thought to be a puzzle or a mystery (Sterelny, 1997; Alexander, 2008; Fitch, 2010, p. 417), and (3) it is difficult to explain their emergence without assuming that humans developed these traits to survive as a species or “for the good of the group.” It is evident, for instance, that cooperation and altruism can benefit the group, whereas it is much less clear how they benefit individual reproduction. The same is true for language. Linguistic communication can be used to share knowledge, to exclude cheaters by spreading gossip about them, and to plan well-coordinated actions (Nakamaru and Kawata, 2004; Giardini and Conte, 2012; David-Barrett and Dunbar, 2016). All of this would give an advantage to the group. At the same time, it is well established that complex traits cannot evolve simply because they benefit the group or the species (Hamilton, 1964; Williams, 1966; Mayr, 1982). In this paper, I will try to solve this contradiction.

### Two Criteria for a Theory of Language Evolution

Derek Bickerton (2007, and Bickerton and Szathmáry, 2011), who has spent about 40 years researching the origin and evolution of language, has suggested that any theory of language evolution should meet two criteria: (1) it should not only describe the evolution of language but also explain people’s extraordinary willingness to cooperate with non-kin and other forms of altruism (costly behavior performed by an individual that increases the proliferation of its group)^1^. (2) A sound theory of language evolution should describe the specific and unique selective pressures under which the human language faculty evolved in one line of apes but not in others.

These are the rationales behind these claims: (1) it is widely acknowledged that both language (Tomasello, 2003; Chomsky, 2006) and human altruism (Alexander, 2008), including cooperation with non-kin (Bowles and Gintis, 2003; Sperber and Baumard, 2012), are expressed in humans uniquely among primates. In the rather short evolutionary time span of approximately 6 million years during which our ancestors evolved, after splitting from the ancestors we share with chimpanzees, it would be—according to Bickerton and Szathmáry (2011)—remarkable enough to have developed even one unique trait found nowhere else among life on earth. That two or more such traits developed independently in one species seemed highly unlikely.

Bickerton and Szathmáry (2011) also point out that both problems—the puzzle of language evolution and that of human cooperativeness—are closely related in a virtually paradoxical structure:

*Could co-operation have led to language, or vice versa? There are problems with either solution. A “language-first” model faces the difficulty that language presupposes a level of trust unlikely to exist given the conniving and deceit found among non-human primates (Whiten and Byrne, 1988). Why would anyone believe verbal utterances, given that words are such “cheap signals” (Zahavi, 1993), and how, if no one believed them, could language have taken root? However, a “cooperation-first” model faces an equal difficulty in that most evolutionary studies of human cooperation assume the existence of communal norms and the punishment of infractors (Henrich and Boyd, 2001; Fehr et al., 2002; Bowles and Gintis, 2003). It remains unclear how such norms could have been established without any kind of language.*

From this Bickerton and Szathmáry deduce that cooperativeness and language must have the same evolutionary history and thus should be explained by a single theory describing their coevolution (cf. Zlatev, 2014).

(2) The second criterion is based on the idea that if “any adaptation is unique to a species, the selective pressure that drove it must also be unique to that species; otherwise, the adaptation would have appeared elsewhere, at least in rudimentary form.” (Bickerton, 2007, p. 514). This entails that a model of language evolution should explain not only why our ancestors^2^ developed language but also why other great apes did not.

This claim is based on an understanding of the evolutionary process that surpasses the more conventional idea that traits always develop as an adaptation to interactions with the natural environment of a species—leaving aside all well-documented structures in which a species creates its own selective pressure, as described in concepts like *cognitive niche construction* (Tooby and DeVore, 1987), *co-evolution* of culture and brain (Deacon, 1997), *sexual selection* (Miller, 2000), or the *social brain hypothesis* (Humphrey, 1976).

Human evolution includes some stages that seem unusually rapid, compared to most examples of evolutionary development (Pollard et al., 2006; Prabhakar et al., 2006; Bush and Lahn, 2008; Britten, 2010; Hughes et al., 2010; Burbano et al., 2012). One indication of this unusually rapid evolution is that the brain size of our ancestors tripled within roughly 3 million years and the cognitive abilities of *Hominini*^3^ probably developed with extraordinary rapidity (Lahn et al., 2004). Lahn suggests that “to accomplish so much in so little evolutionary time—a few millions of years—requires a selective process that is perhaps categorically different from the typical processes of acquiring new biological traits.” If this is true, human evolution should probably be considered to have involved a *major transition in evolution* as proposed by Maynard Smith and Szathmáry (1995). Other examples of *major transitions* concern the emergence of eukaryotes, of multicellularity, of sexual reproduction, and of colonies of eusocial insects, each of which fundamentally changed the “logic of evolution.”

Maynard Smith and Szathmáry identified several properties common to these transitions in the evolutionary process, such as smaller entities forming larger entities and differentiating themselves functionally while sharing their resources. Such entities can sometimes fall back into selfishness (e.g., the replication of selfish genetic elements, cancer, parthenogenesis). In general, the pace of the evolutionary process and its complexity increase (immensely, for example, in the transition to sexual reproduction) and often the manner in which information is transmitted is modified.

Maynard Smith and Szathmáry (2000, S. 139) claim that language is the foundation of the major transition in human evolution because language alters the transmission of information and makes cumulative culture possible. In contrast or addition to this view, I suggest that a much earlier major transition concerning human evolution brought about the unique evolutionary system in which humans and language evolved and that the foundation of said transition is the result of *equilibrium selection.*

*Equilibrium selection* refers to a two-level evolutionary system. On the lower level, agents adapt to *the best individual strategy*, while on the higher level selective processes occur between different equilibria (populations of agents), which in most cases cannot be foreseen by the agents. Unlike with *naïve forms of group selection*, there is no magical connection between the selection among equilibria and the strategies of the agents, because agents in this model can only act in their own reproductive interest.

The pivotal idea introduced in this paper concerns the emergence of a *backchannel* between the two levels of selection. I show by modeling that a backchannel—allowing information from the higher level to affect selection at the lower level—will, under certain circumstances, develop naturally if equilibrium selection is in place. I also suggest that these circumstances apply to human evolution. Examples of systems utilizing a backchannel are nerve cells being better nourished when used more frequently, enabling them to be conducive to the survival of the whole organism, or a law firm in which all employees get paid to the extent that they contribute to the survival and success of the firm. In both cases, the selection on the higher level informs the selection on the lower level. I promise that all this will become much clearer soon.

### The Concept of Evolution Used in This Essay

The hypothesis suggested in this paper is not committed to any particular version of evolutionary theory, though I decided to use, in this first sketch of the model, the most conservative and strictest version of evolutionary theory for strategic reasons: if I can defend my proposal in terms of this theory, it will be also valid for all other extended versions of evolutionary theory, which, roughly speaking, would make the explanation easier and the adaptation process faster (for example, by adding more hereditary mechanisms, such as epigenetic inheritance, or by emphasizing ontogeny and social learning). I identify this most conservative and strictest version of evolutionary theory as what is sometimes called *the standard theory* (an up-to-date version of the *modern synthesis*) which defines evolution as change in allele frequencies within a population and predicts adaptation to optimize the reproduction of each individual, (Hamilton, 1964; Williams, 1966; Mayr, 1982), without assuming that natural selection can “directly see an individual organism in a specific situation and cause behavior to be adaptively tailored to the functional requirements imposed by that situation” (Tooby and Cosmides, 1992)—as sometimes defended by advocates of the Gene’s Eye View.

Please note that this strategic choice is far from an ontological claim about the nature of evolution and that the hypothesis presented in this paper is compatible with any extended version of the modern evolutionary synthesis—even with those that are unlikely to be true.

### The Structure of the Argument

In the following part of this essay, our hypothesis will be developed in three steps. First, I will outline for the sake of example a simplified model to illustrate the overall development I am proposing. Second, I will discuss the biases and obstacles of this development from the perspective of the individuals involved. And third, I will discuss the dimensions of uncertainty. Finally, I will briefly discuss the explanatory power of the model. This is the plan in more detail:

In section “The Toy Model,” I will present a thought experiment in the form of a toy model to project the overall development of an equilibrium selection. This will be done in two steps. First, I will explain why our ancestors—but not the ancestors of other apes—became subject to equilibrium selection and then I will try to show how and why, in this case, a backchannel naturally emerges.

The function of the toy model is to predict the state toward which the system tends to evolve: the *attractor of the system*. It is a *toy model* because it only exemplifies the main idea by postulating the *existence* of a backchannel between the two levels of an equilibrium selection by arguing for *one* concrete possibility. In other words, the toy model expresses the hypothesis that *at least one such mechanism exists*—and that thereby the problem of cooperation/altruism can be solved—without implying that this is the *only* mechanism.

In section “The Noise That Finally Improved the Channel,” the proposed development is analyzed in terms of individual strategies. I shall show that the system with two levels of selection and a backchannel described in the previous section will naturally develop a bias, which can be interpreted as noise in the channel, and that this bias will at the same time improve the backchannel in the long run by putting many cognitive capacities under selective pressure.

In the section “Biases, Uncertainty and Explorative Power,” I will adapt the simplified model to a much messier reality by doing three things: first, I will investigate the proposed development in terms of the logic of optimal reproduction of each sex. Second, I will discuss the dimensions of uncertainty and extend the model by discussing functional equivalents to the mechanisms suggested in the toy model. Finally, we will briefly examine the explanatory power of the model and discuss how its predictions can be empirically tested.

## The Toy Model

In this section, I will suggest a simplified model in which a climate-changing geological event initiates a process in which apes—with a chimp-like brain size living in a multi-male/multi-female group—set off on their own evolutionary trajectory heading toward what humans are today.

### Three Necessary and Jointly Sufficient Conditions for Equilibrium Selection

Since the model introduced in this paper suggests that evolution of language and cooperation in humans can be explained in terms of equilibrium selection, I shall explain how and why humans, but no other apes, became subject to such selection on two levels. The condition for any entity to become subject to equilibrium selection is that the higher level of selection—in this case groups—becomes a target of adaptation by natural selection (Boyd and Richerson, 1990).

To become a target of adaptation by natural selection, the entity in question—in this case groups—needs to meet three qualities: they need to be diverse, there need to be selective processes going on between them, and the mechanisms on which the fitness differences are based need to be stable enough to be passed on. These correspond to the three necessary and jointly sufficient conditions Darwin (1859) claimed for evolution to work: variation, selection, and heredity.

It has been pointed out (Boyd and Richerson, 2009) that many primate groups, including groups of chimpanzees, meet two of the three conditions: they vary and selective processes are continuously going on among them (Goodall, 1986; Mitani et al., 2010). However, what is lacking is *heredity*—the passing on of cultural or genetic variations of the group to descent groups. Culture does not vary much between descent groups of chimpanzees because chimps lack fast-developing cumulative culture^4^ and genetic differences between groups are not conserved because—at least in the rainforest—females often mate with males from other groups (Gagneux et al., 1999) and also migrate to other populations.

Boyd and Richerson (2005, 2009) argued that to develop stable differences that can be subject to selection on the group level, the developments within each group need to be fast compared to the level of intermingling between groups. In other words, to explain how our ancestors became subject to equilibrium selection, we must pinpoint either an increase in distinctive developments in each group—producing heritable differences between groups—or a decrease in intermating between members of different groups.

In the next subsection I will suggest how groups of our ancestors might have become more or less reproductively isolated and explain that our very existence may be founded on a geological event.

### The Climate Change East of the Great Rift Valley

In the last 30 million years, the Great Rift Valley, reaching from today’s northern Syria to Mozambique, was formed by extraordinary tectonic forces. The mountain ranges on each side of the valley (itself around half a mile above sea level) rose further (up to two miles) due to volcanic activities about 10 million years ago—a geographical event that caused the prevention of moist air from the west passing over East Africa. At the same time, a shift in weather patterns was causing the whole African continent to dry out. This caused the topography of East Africa to completely change from rainforests to savannas—mainly consisting of gallery forests and grassland (Coppens, 2004).

There are many theories on how this significant climate change was responsible for humans evolving. One previously common theory is that the adaptation to the savanna made our ancestors bipedal and collective hunters of game (e.g., Ardrey, 1976). In contrast to this suggestion, I shall claim that, due to the climate change east of the Great Rift Valley, groups got more or less reproductively isolated and thus became the subject of adaptation by natural selection.

To understand the effect of the new habitat on our ancestors we need a rough picture of how they lived. Let us conjecture that the region was inhabited by a number of hominid populations, anatomically similar to the last common ancestors of chimpanzees and humans. It has been observed that rainforest chimpanzees actively seek mating partners outside their social unit (Gagneux et al., 1999) though these males might be killed when caught in rival territory (Mitani et al., 2010). Territories are guarded by the males of each group, usually by patrolling their respective boundaries (Watts and Mitani, 2001). We should also take into account that the territories in savannas are about twelve times larger than in the rainforest (Hunt and McGrew, 2002) and that trespassers would need to cross open grassland, leaving them exposed.

In this subsection we discussed how a change in climate might have reduced the intermating between groups. In the next subsection we will discuss two mechanisms that can rapidly produce heritable variation on the group level.

### Sexual Selection and the Extended Founder Effect

Sexual selection (which is an important selective force in chimpanzees, Stumpf and Boesch, 2006) has five characteristics that particularly support the production of heritable differences between groups:

(1) Sexual preference of females is a selective force on the individual level, influencing the evolutionary process. Female preference can be passed on to splinter groups, while this would be, for instance, impossible with other factors of group fitness, such as advantages based on the immediate environment or group size.
(2) Sexual selection can influence the best evolutionary reproductive strategy of both sexes (Miller, 2007). In the case where costly behavior is the target of sexual selection, it can produce different predominant *evolutionarily stable strategies* for the chosen sex (Nash equilibria) across different groups, thereby producing variations in group fitness, which then can be—under certain circumstances (see section “Three Necessary and Jointly Sufficient Conditions for Equilibrium Selection”)—selected on the group level.
(3) Sexual selection is a strong force and can be faster and more precise than adaptations to interaction with the natural environment (Miller, 2000).
(4) Traits acquired by sexual selection are generally passed on to both sexes. Dimorphism only emerges when the trait is costly but not beneficial for the sex with the greater parental investment (Trivers, 1972)—typically the female. In this case its expression is epigenetically controlled.
(5) Sexual selection can emerge by chance, and then—if it does not conflict with other forms of sexual selection already in place—escalate in a runaway process (Fisher, 1930). This is because it is possible that new forms of female choice can spread, piggybacking on the selected trait that is circulating throughout the population (Jones and Henshaw, 2019): When a new non-conflicting female preference emerges, the males with the preferred trait gain a reproductive advantage because they can mate with all the females that any other male can, but additionally have an advantage with the females who have the new preference. Now the trait itself and the preference will correlate due to the fact that males having the trait and females preferring it will mate with each other more frequently. As a result, the preference and the preferred trait might spread through the population.

In a population in which female choice is the strongest evolutionary force, what will eventually turn out to be the best female choice is dependent on, among other things, the choice of the females in the next generation. In other words, the quality that females should choose in a male is his attractiveness because this increases the likelihood of having attractive sons, therefore increasing the likelihood of the sons being chosen as well—ultimately increasing the likelihood of the choosing females’ genes to prosper and have many grandchildren. This effect—sometimes called the *sexy son hypothesis* (Weatherhead and Robertson, 1979)—makes it possible for different groups in the same habitat to develop differently due to sexual selection (Lande and Kirkpatrick, 1988).

If female choice only differs slightly between groups, the groups might develop very differently, often in an unpredictable way (Miller, 2000). This process of differentiation between groups based on different predominant sexual selection pressure is strengthened by the *founder effect*: when various groups split off from the same parent group, the likelihood that they deviate from each other by chance is inversely proportional to their size (Mayr, 1942). In the case of our ancestors, it would be reasonable to assume that this random effect of producing genotypical and phenotypical differences between descent groups due to the founder effect would also apply to female mating preferences.

In addition to the general *founder effect*, sexual selection could also play a role in *producing* new groups. It seems likely that individuals with certain mating preferences and their sexual partners who show the desired features would split off together. Assortative mating (individuals with similar phenotypes being more likely to mate with each other, Jiang et al., 2013) would also contribute to producing these splinter groups, increasing similarity *within* each splinter group and thus increasing the differences *between* them. For all these reasons, the variability between groups (including the female mate preference) is higher than it would be if a random group split off. Call this *extended founder effect*, which could have played a role when groups of our ancestors split off from their parent group, when a limited territory of gallery forest and its surrounding grassland no longer provided enough resources for the whole group.

### A Model to Illustrate the Selective Processes on Two Levels

I argued that equilibrium selection could emerge when intermating between groups is low compared to the distinctive development in each group. In the last two subsections I defended the view that this was the case in the *Hominini* line. I suggested that one possible motor of this development was the predominant female preferences in each group choosing different mating partners and thereby producing heritable variations on the group level.

Later we will see that these differences between groups can be produced by any social form of selection. The main point is that these differences include the framework for further development within each group by creating various selective pressures that establish different equilibria. Now, I shall defend the view that the equilibrium selection described will naturally produce a backchannel between the two levels of selection. I will illustrate this by a *thought experiment*.

Suppose in one group, females like long noses. Since long noses are considered attractive, it is a good strategy to choose mating partners with long noses because their offspring are likely to have attractive long noses. In another group, say, females choose good hunters as mating partners. Now, everything else being equal, it seems likely that the hunter group would displace the long noses, for two reasons: first, the group in which the females prefer good hunters will probably be better at exploiting the territory. Second, this group will be better equipped for conflicts, because genetic, ontogenetic, and cultural adaptations for hunting contribute to physical strength, tactics, and coordination between group members, which can all be utilized when it comes to intergroup conflicts. The result will be that the group in which good hunters were bred by female choice will expand at the expense of the long noses.

Let us assume that different descent groups splitting off from the surviving parent group differ again in their predominant female preference. They both favor good hunters, but they differ in how they choose them. In group A the females choose males that provide them and their offspring with tasty and nutritious food. In group B females prefer males that hunt dangerous or hard-to-catch animals. In this case, it is not really clear which group will survive. On one hand, the group in which the females choose males that feed them and their offspring would probably reproduce better than the group whose females mate with males that kill dangerous and hard-to-catch animals. On the other hand, the latter group will improve their hunting skills and thereby their capacity to defend themselves faster. Which group would survive might become a matter of circumstances.

What I would like to show now is that both groups have the potential to develop into a group that would displace all others by developing a backchannel:

Group A: Female choice needs to be selective. Females that choose the good meal will have the opportunity to be selective only if not all males have the ability to feed them and their young. If all or most the males in the group do meet this minimum standard, a good female strategy would be to choose males that bring them more food than they and their kin can consume. It will, in all likelihood, be tolerated that the surplus will be eaten by non-kin. A good female choice would be to choose males according to the amount of surplus they produce.

Group B: The group in which females choose good hunters could further split into two groups. In one group males might display their hunting success by natural signs such as hunting trophies. In the other group they might display their hunting ability by sharing the food with everybody. Both possibilities are equally good from the individual perspective of the choosing female when it comes to the proliferation of their genes in the gene pool of the population. However, groups in which the kill is shared with all group members will probably end up displacing other groups.

In the end, both hypothetical groups A and B will become groups with a more or less similar structure: in both cases females choose males that display and somehow share the kill.

We defined a backchannel as a mechanism that makes it possible for the lower level of selection to be informed by the higher level of selection. This is the case here. A social form of selection—in this case, female mate choice—gives rise to prosocial behavior. If such a group were to emerge, it would give the group an immense advantage over all other groups. It would also change the social structure of the group fundamentally enough to speak of a major transition in evolution.

In this subsection I tried to illustrate what it takes for a backchannel to emerge between two levels of equilibrium selection. If, for instance, females were to choose hunters that share the meat of their kill with non-kin—a behavior that is beneficial for the survival and proliferation of the group—this would increase the reproductive opportunities of the males displaying the objectively prosocial behavior. This exemplifies the previously given definition of a backchannel as a complex mechanism in which the lower level of selection (the change of the allele frequency) is informed by the higher level (the selective processes between groups). When this backchannel is in place, individuals adapting to behavior that improves the reproduction of their genes will develop the congruous prosocial behavior.

We also saw that there are different possibilities for a backchannel to emerge. These examples were given to illustrate the contingence of the development. There is no necessary development in each group to create a backchannel. However, if a backchannel were to emerge in one group, this group would probably displace all others. In the next subsection I shall explain why this is the case by changing the perspective to a more abstract view.

### The Attractor of the System

In the example given in the last subsection, each group develops according to the prevalent female preference, choosing certain forms of behavior and appearances, while penalizing others. This shapes the social structure, influences how individuals develop in their lifetime, and puts selective pressure on the qualities in question. In a second step, groups compete for territory. What we should see is a dynamic adaptive process on two levels, in which the female preference will adjust according to its ability to produce groups displacing all others. Since the *extended founder effect* repeatedly creates variations at the level of the predominant female choice, the system is unstable and prone to escalation.

One might ask, is there always a better female choice criterion possible or is there a best one that, once established, can no longer improve? I will try to show that, although in reality the process of optimization will continue, there exists a theoretically best female choice criterion that can be formulated abstractly. This abstract formulation of the best possible female choice corresponds to the *attractor* of the system. Although an attractor defines the direction of the movement or development, it is not guaranteed that the attractor can ever be reached. Since, in our model, the development of the groups depends on the female choice criterion, the attractor of the system must be defined in terms of the optimal female choice criterion. This suggests that the relevant dimension of the female choice criterion creating different equilibria in various groups concerns the capability of each equilibrium to influence the group’s ability to displace other groups.

Thus, we might say that the actual object of selection between groups is *the prevalent female choice criteria in each group, which compete in their ability to create groups that displace all others*, although this does not specify which qualities females actually need to choose to meet this criterion.

Miller (2007), for example, suggests that females should choose males that are kind, friendly, reliable, and able and willing to invest into their future offspring. A male should be brave and heroic in defending family members and their rights and resources: a tender defender (Tucker and Miller, 2015). This would be a good choice indeed. It is a strategy that would benefit the individual female. It is exactly the right answer to the question of rational choice on the individual level, informed by how selection actually works. But it would be not the female choice that could transform the gene pool in such a way that the group could displace all other groups. For instance, groups in which everyone aims to win wars against other groups would probably displace groups consisting of members that only protect their own families. We must remember that we are not asking for a realistic solution (which would have to be evolutionarily stable on the individual level), but for an ideal scenario to predict the attractor of the system. The answer I want to suggest is: The ideal scenario would be *females choosing males according to their contribution to the proliferation of the group.* This could also be described in terms of the best possible backchannel between the two levels of selection.

In this subsection we have discussed the attractor of the system: female choice according to the male’s contribution to proliferation of the group. Proliferation can mean two things: exploiting the resources of the territory more efficiently or expanding the group’s territory. The exploitation of the savanna territory is greatly improved by hunting skills. The expansion of territory can apply to inhabited or uninhabited territory. Since space is ultimately limited, expansion into uninhabited territory is necessarily temporary. This suggests that the best female choice would be to choose great hunters and good defenders of the territory. However, there is a crucial technical limitation with either scenario: it requires an information channel between the prosocial actions of males and the cognition of the choosing females.

### The Media of the Backchannel

We saw that the attractor of the system would be females choosing males according to their contribution to the proliferation of the group. This requires females to observe the behavior they would be basing their choice on. In many cases this is impossible. Conflicts between groups of chimpanzees, for instance, typically take place at the borders or even within the territories of neighboring groups (Nishida and Hiraiwa-Hasegawa, 1986), while most females stay with their offspring near the center of their territory or in a secure space (Pepper et al., 1999) and often do not engage in territorial conflicts (Wrangham and Glowacki, 2012)^5^. This is to say, an information channel is required for a backchannel to evolve—in this case, between the theater of war and the feeding and breeding areas.

Let us imagine that the goddess Athena were to have revealed the deeds of the males to the women in their dreams; then this would have been a perfect information channel between the behavior of the males in conflicts and the mate-choosing cognition of the females. Alternatively, we could imagine a medium consisting of small video cameras implanted in the skulls of males that transmit wirelessly to portable screens carried by females.

Unfortunately, we are talking about our apish ancestors and therefore about a time in which neither Greek gods nor video cameras were around. At this point I invite the reader to brainstorm what kind of media could be established that is able to transmit the behavior of the males hunting or engaged in territorial conflicts to the breeding areas. The answers given in this paper are not meant to be exhaustive and it would be of great help for this project to list all possible media that could enable transmission of differences in behavior during hunting or group conflicts into differences of reproduction.

One medium that presents itself, since it has the ability to transmit information about non-present events, is *language in the form of storytelling*. Please note that this is still a thought experiment. For a realistic evolutionary narrative, we must distinguish between two claims: that a group that developed storytelling and used it as a backchannel would have, all other things being equal, displaced all other groups; and that language and storytelling developed *because* it would have helped the group to displace others. While the first claim is one of the main arguments of this essay, the second claim is flawed because complex traits can only develop when different phenotypes compete for reproduction and not for the sake of the group. That the genetic basis of storytelling is complex is suggested by the fact that storytelling needs many different cognitive abilities, including language, theory of mind, and episodic memory, that are uniquely expressed in humans. A flawed argument would be: our ancestors could only survive by developing storytelling, thus they did. However, the strategy of this paper is that I first outline the ideal trajectory from the perspective of the group and only later discuss whether and how such a development would have been possible, assuming that any evolutionary development has two conditions: first, it has to be driven by individuals gearing toward reproduction of their genes (as a result of a competition for reproduction, avoiding fitness teleology), and second, it needs to be a more or less gradual process on the gene level (Williams, 1966)—yet not necessarily on the level of the system where transitions, as we saw, sometimes occur.

What we would need to find to satisfy the criteria of gradualism would be a much simpler medium from which language and storytelling later developed. Such a simpler medium would be, for instance, a natural sign that represents a past action. An example of such a medium was already provided in subsection “A Model to Illustrate the Selective Processes on Two Levels,” where natural signs—in this case, displaying a kill and food sharing—were suggested as media for communicating past actions.

In this subsection we discussed the required information technology for a backchannel to emerge. We found that a good medium would be telling stories about hunting or combat behavior. A more primitive medium, with much lower resolution, would be natural signs, such as the presentation of kills. In the case of intergroup conflicts, for instance, trophies made from body parts of the enemy could be carried over a distance to be displayed. This could be considered propositional communication. The prediction is that the backchannel will improve itself over time because groups with a better backchannel will displace other groups. This claim is independent of the means by which the backchannel is implemented. At the same time, we said that we cannot infer that the group’s benefit from storytelling led to the development of storytelling, because the reason for the evolution of a complex trait cannot be solely that it helps the group’s survival but needs to be explained in terms of individual selection.

### How Humans Became the Most Dangerous Animal

We said the proliferation of a group would depend on the size of its territory or how that territory is exploited. Since ultimately space is limited, expansion of territory is bound to be conflictual. If the main factor of the group’s success depends on exploiting territory and feeding the group, the female choice of good hunters that share their kill with the group might be the attractor of the system. If, on the other hand, the main factor of the success of the group depends on populating new territories, the females should choose males that make this possible. If the spread of the group includes displacement of other groups in raids, then the attractor would be females choosing males according to their contribution to expansion through conflict, or, more simply: how a male contributes to victory over other groups.

There is an argument for why trophies inevitably become war trophies. Say, at a certain point, a bear proves to be the hardest animal to hunt and more dangerous than any other animal, including members of other Hominini groups. When females choose males according to their hunting performance, represented by trophies, those hunting abilities may improve, including innate talent and perhaps simple weapons and other forms of hunting culture. Thereby we should assume that—some thousand generations later—any animal, including the bear, would be much easier to hunt. Now the other Hominini groups, in contrast, may experience the same improvement in their hunting abilities and thereby increase their overall threat and eventually become more dangerous than the bear. While the hunting of all kinds of animals will eventually become a manageable task as hunters’ abilities improve, other tribes will remain dangerous. The selectiveness of female choice therefore predicts that, at some point, war trophies will be more appreciated than hunting trophies, thus giving war heroes a higher reputation than good hunters.

The dynamic of the displacement process is enforced by the fact that war-like conflicts in small-scale societies often take the form of raids. Raids give a major advantage to the attacking group (Wrangham and Glowacki, 2012), an advantage that increases with improved tactics, coordination, and weapons. This makes *strike first* the best strategy.

In the further course of this essay, I will add more and more layers to the model. We will see, for example, that after language develops the backchannel functions increasingly as a long-term storage medium (in addition to being a transmission medium): the reputations of every individual are stored in the memory of storytellers and audience and are distributed in circulating narratives. At that point, the backchannel does not build up the reputation of an individual from scratch but only influences the preexisting image. We will also see that sexual selection does not need to be direct but can rely on a much more complex female choice, including the social status of males built on circulating narratives or natural signs of prosocial actions such as trophy display of kills, whose meat is later shared.

In the next section we will discuss a main obstacle that hinders the development of a perfect backchannel (the translation of male contribution to the proliferation of the group into reproduction of the contributors), which is based on the differences in males’ ability to communicate their past actions.

We shall also see that the communication of one’s past actions can produce major selective pressures on the cognitive capacities of both sexes and thereby improve the resolution of the backchannel in the long run.

## The Noise That Finally Improved the Channel

### Noise and Bias

In our model, the terms *noise* and *bias* may refer to the same phenomenon from different perspectives. *Noise* is understood to be anything that hinders the perfect translation of prosocial behavior into reproduction, considering only the strength of the corrupting force. *Bias*, in contrast, includes *how* the signal is distorted. Understanding the bias often allows us to predict how the system will develop.

In the last section, I tried to reconstruct the overall development of the system from the most abstract perspective possible. In this section I will explore one of the main biases. I shall try to show that this kind of bias can improve the quality of the channel in the long run. To show this, I will add an essential aspect of the individual strategies to our toy model and then resketch the model to capture the overall development in more detail. Please keep in mind that I am still outlining a simplified model, to which I will now add another layer: how the backchannel gives rise to a certain form of exploitation that is likely to escalate.

We shall see that one of the main sources of noise lies in the variation in the ability of males to communicate their past actions. A good storyteller, for instance, is more impressive than a poor storyteller. The same is true for an imaginative presenter of trophies. Thus, in some cases, a good presenter of past actions might be more attractive despite having undertaken less extraordinary exploits than his competitors. This obviously hinders the perfect translation of prosocial behavior into reproduction. At the same time, the bias produces a major selective pressure under which many unique human cognitive abilities may have developed. In other words, it is suggested that without understanding this bias, the evolution of essential aspects of human cognition—including language—would stay a riddle.

### The Logic of the Female and Male Strategies

In multi-male/multi-female groups, every individual competes for reproduction with every other individual of the same sex (especially with non-related individuals). We saw that in a population in which female choice is a major force of selection, the reproductive success of the genes of every female depends on the future female choice (e.g., regarding the attractiveness of her sons), while the reproduction of males depends on the present female choice and the competition with other males to be chosen (Weatherhead and Robertson, 1979).

When sexual selection is in place, males will exaggerate the qualities chosen by the females to maximize their reproductive success. How this plays out depends on the nature of the chosen quality and how it is assessed. Some qualities are reliable indicator of fitness and easily evaluated; for instance, deleterious mutations affecting body symmetry or perceptible parasite infections. Other less reliable traits are reinforced to the point that their expression carries enough costs to be reliable. Such costly traits (for example, the long and colorful trains of male peacocks) create sexual dimorphism, since females develop mechanisms not to express them. This is traditionally understood as the *handicap principle*: a trait develops because it is costly and thereby proves that it is a reliable indicator of fitness (Zahavi, 1975).

However, newer research (Penn and Szamado, 2020) has shown that the *handicap principle* is neither a valid simplification nor predictive. Sexual characteristics do not develop *because* they are costly, but instead males will find the best cost-benefit ratio for developing costly traits^6^. This is to say that males strive to optimize their attractiveness at the lowest possible costs.

According to our model, the impressions males communicate to the choosing females depend on two factors: the past actions that are communicated and the quality of the communication. While the past actions are costly, communicating the action is comparatively cheap. At the same time, the effectiveness and quality of the communication of past actions is restricted by the communicative abilities of the male in question, which are in turn limited by his cognitive faculties, among other things. This suggests that the cognitive abilities of individuals to present their past actions (e.g., by trophy display or first-person storytelling) will undergo strong selective pressure via female choice.

From the perspective of the system, the ability to effectively display trophies or tell stories is, on one hand, a necessary condition for the channel to work; but, on the other hand, when unequally distributed across the performing males, it can be considered noise, hindering the unbiased translation of the prosocial actions into reproduction.

I will try to show that this noise is likely to increase within each group. The argument is that females paying a little more than average attention to the performative abilities of males will be, all other things being equal, reproductively more successful than other females. The reason for this is that representing a past action well—for instance, by skillful trophy presentation or impressive storytelling—comes with fewer risks than actually performing heroic deeds. This is relevant for the choosing female with regard to her potential sons. When the attractiveness of their sons is based mostly on performative qualities, the sons do not need to risk their lives as frequently, resulting in better survival of their (and their mother’s) genetic material. The same is true for the male mating partner. If the male mating partner puts his life at risk less often, he is more likely to survive, which is relevant if he invests time, energy, and resources in the common offspring. Therefore, it is a good female strategy to be a little more impressed by male performance than the average female in the population. Since this is true for all females, the choosing females will tend to take male performances (such as trophy display and storytelling) increasingly into consideration during mate selection.

### Selection Between Groups

In the last subsection I explained why groups might have the tendency to mold males into great presenters of their past actions. From the perspective of the system, female preference for narrators and self-promoters is noise in the backchannel. The weakening of the reliability of the backchannel makes the groups less aggressive because the males have less ambition to produce trophies or brave deeds that can be told.

For better clarity, I will now simplify the manifold reality of different groups to two highly idealized classes: (1) groups in which the typical female is more fascinated by the representation of past actions and (2) groups in which the typical female cares more about the prosocial action presented by physical evidence or verified stories. Call the first *storytellers* and the second *warriors*.

Imagine a habitat in which all groups have the tendency to slowly transform from warriors to storytellers. The pace of this process might differ between groups. In general, all other things being equal, *storytellers* will be displaced by *warriors* on the group level. This displacement process does not necessarily mean that all members of the other group are killed (Richerson and Boyd, 2005).

It was found that young females, in particular, are frequently integrated into victorious groups (Chagnon, 1968; Annan et al., 2011). The genetic diversity of the victorious group increases through the integration of the alleles from the displaced group. It is therefore likely that the genes encoding narrative abilities in the defeated storyteller group, which are not present in the warriors, are transmitted from the storytellers to the warriors (for instance, by the captured storyteller females to their warrior sons). This transmission requires that the new alleles be present at least once in any of the integrated individuals.

Given this presence, it is likely that these alleles—improving the narrative talent—will spread in *any* group, including the groups of warriors, because they generate an individual reproductive advantage at little cost. The effect of this dynamic is that any beneficial alleles in any group concerning the ability to represent past actions, including narrative skills, can be incorporated into the victorious group.

This simple model illustrates the suggested process: within each group there is a slow shift in female preferences in favor of great storytellers. This shift in female choice, which focuses increasingly on narrative skills and decreasingly on heroic deeds, causes the group to lose aggressiveness and the ability to displace other groups. Groups in which this process is faster are therefore displaced, all other things being equal, by groups in which this process is slower. Now, what does this mean for the development of the system as a whole?

One could argue that if any single group develops toward storytelling, the whole system needs to shift toward storytelling, because the whole system is just another word for every single group. The counterargument would be that generally storytellers are displaced by warriors and that it would take only one extremely aggressive warrior group to displace all others. In other words, there are two opposed forces: the tendency to shift toward storytellers and the ability of warriors to displace storytellers. In which direction the whole system finally develops depends on the pace in which warriors’ groups are molded into storytellers and the ability of the system to produce variations along the warrior–storyteller continuum.

The ability to produce variations of the female choice criterion that governs each group depends greatly on the extended founder effect. Males with particularly well-developed self-expression and storytelling skills and the females that prefer storytellers attract each other; likewise, the female individuals that prefer heroic warriors will be attracted to the object of their sexual desire. Thus, they are likely to split off together, when the limited gallery forest and the surrounding grasslands cannot support the size of the group.

In this subsection, I used a simplified model to illustrate how the overall process in a given habitat might have been shaped by two driving forces: the tendency of every group to move toward storyteller on the warrior–storyteller spectrum and the antagonistic force that storytellers are displaced by warriors. We also saw that mutations that improve narrative skills can originate in any group within the habitat and later be absorbed into the surviving group, for instance, through the female germline. While some complex narrative cultures might vanish, the genetic basis of the narrative talents selected by these cultures will, in most cases, spread through the habitat, because they produce a selective advantage without significant costs to individual fitness. At that point, it is only a matter of time before all members of the group share the introduced alleles. The pace of the development also depends on the number of alleles that can improve storytelling—probably at thousands of loci—and the number of individuals in the entire habitat.

In the next subsection I will try to show that every cultural mechanism that tends to block female choice of storytellers over warriors will be selected on the group level. This might include mechanisms to expose lies, the enhanced appreciation of evidence such as trophies, and the recognition of humble heroes who let their actions speak for themselves. We will see that such cultural mechanisms are particularly important for expanding groups, since they integrate individuals from other groups and thereby incorporate the alleles that might encode their stronger preferences for storytellers into their gene pool.

### Cultural Group Selection and Recapitulation

In the last subsection, I tried to demonstrate how the system develops depending on two antagonistic forces: the development of each group toward storytelling and the tendency for warrior groups to displace storyteller groups (based on the ability of the system to produce variations among groups). However, these variations among groups are not limited to genetic differences, such as female preference and adaptation to it, but can—from a certain point onward—include cultural elements. It is likely that cultural mechanisms that block the tendency to soften a population into one of great talkers will be positively selected on the group level. This would be *cultural group selection* (cf. Henrich, 2004; Boyd and Richerson, 2009).

Cultural group selection includes the idea that social norms can be selected on the group level (Boyd and Richerson, 2009). This would predict that cultural mechanisms that foster the reproductive opportunities of warriors and block the reproductive chances of storytellers would be positively selected in group competition. A candidate for a strong natural force against the recognition of the great narrator lies perhaps in the competition between males as they compete for status. They should also be motivated to examine the truth of their competitors’ stories. It may also be possible that storytelling seducers who put their comrades in danger through cowardice are punished.

We can distinguish between conventions securing the standing of heroes, such as rituals honoring them or signs of honor restricted to few, and values that have a similar function. Values are defined here as *transformation rules* converting information about an observed, evidenced, or narrated action of an agent into an imagined essence of the character of a person, often dominated by the dimension of respect vs. contempt. Some of the values might be built on cultural mechanisms such as conventions, institutions, or legends and myths that convey these values and propagate legendary heroes as role models. In our parlance this would simply mean that cultural mechanisms secure the quality of the backchannel and that the attractor of the system can affect both genes and culture.

Before beginning the discussion of the hypothesis, let us recap the core notion of this essay so far. In the second section I outlined the main idea of this essay and extended the concept of equilibrium selection by introducing the idea of an attractor of the system. The main hypothesis I would like to discuss with other scholars is whether equilibrium selection can naturally develop a backchannel between the two levels of selection and whether this was the case in human evolution. The ideal backchannel would perfectly translate the individual contribution to the survival, prosperity, and expansion of the group into reproductive success. The reasons why the attractor of the system can never be reached are the subject of the third and fourth section.

In this third section I tried to show how a bias created by the female preference for good presenters of non-present actions and events will in the long run refine the backchannel by improving storytelling abilities in both sexes. I claimed that female choice is influenced increasingly by the storytelling abilities of males and the beauty of their speech. This development, on one hand, distorts the direct translation of the prosocial acts of males into reproductive success. On the other hand, this biased selection puts selective pressures on cognitive abilities relevant to storytelling, including tracking the attention of the audience, developing a theory of mind and episodic memory, and the ability to learn linguistic conventions, understand syntactical structures and acquire a large lexicon. We saw that these qualities will eventually improve the backchannel because they improve the resolution of the channel—as the linguistic faculty and the ability to tell stories improves, the audience gets a better picture of what happened. Precise language is also a better instrument for evaluating whether statements are true.

## Biases, Uncertainty and Explorative Power

Up to this point, I presented a hypothesis about how language and cooperation evolved as elements of a system. This was done in a rather abstract way by presenting a simplified model, which included two forms of selective processes and a backchannel between them. Now we shall switch to a more detailed picture in which all dynamics must be explained in terms of individual strategies adapting for optimal reproduction.

### Biases and Obstacles

In this subsection we will discuss the female bias of choosing other qualities in males aside from their heritable qualities (4.11). Then I will remind the reader of the asymmetry of reproductive success as the foundation of any evolutionary process and show how the female’s need for parental investment might soften the selective pressure and slow down the development (4.12). Finally, we will discuss the bias of the communication medium, which shapes male behavior, female choice, and the development of the system as a whole (4.13).

#### The Logic of the Female Strategy

**FIGURE 1:**
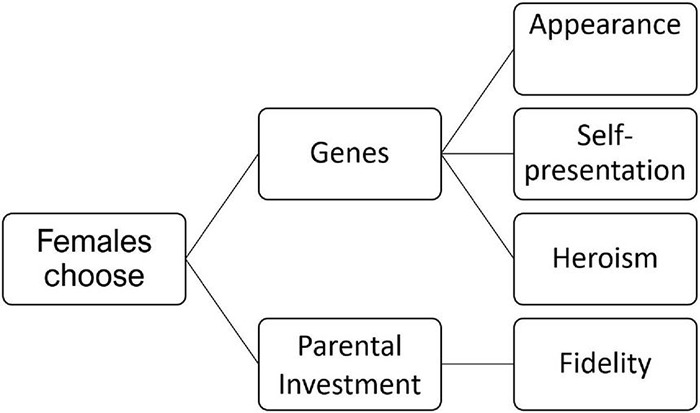
Four classes of female choice. Beside choosing heroism (which explains altruism, see section “The Toy Model”) and self-presentation (which explains the evolution of language, see section “The Noise That Finally Improved the Channel”) there are two more classes which slow down the processes described in sections “The Toy Model” and “The Noise That Finally Improved the Channel”: choosing appearance (selecting health and beauty) and choosing fidelity and kindness (selecting parental investment).

So far, we have assumed that female choice is mostly affected by the quality of the genes being inherited by their future offspring and we have not taken into account that other properties of males can also be chosen by females, including how males behave toward the female and their mutual offspring. In some circumstances it might be better to choose a faithful and kind mating partner who shares all his resources exclusively with his female and invests in their common offspring, rather than to procreate with a high-status male with excellent genes who will never be around. Thus, females are likely to not only choose “good genes”—genes which are prone to increase the fitness of their offspring—but also take parental investment into consideration (Figure 1). Here we ignore compatibilism—which is especially important regarding the immune system—because this dimension is not necessary to explain the overall development.

Choosing the nice guy and a good father of one’s children has many disadvantages for the group. One is based on the fact that the interests of the group (tribalism/altruism) and those of a family unit (nepotism/egoism) often clash (Boyd and Richerson, 2005). Another disadvantage is that niceness might be less costly than heroism and thus less selective. Many males may reproduce by promising fidelity and parental investment. We shall discuss this problem of relaxing selective pressures in the next subsection.

#### The Asymmetry of Reproductive Success

For evolutionary processes to take place, different individuals must have different reproductive success. I said that the optimal backchannel would convert prosocial behavior into reproduction. However, we did not discuss the *exchange rate* for this transaction. The value of this exchange rate is essential for the overall development and the motivation to behave prosocially. If, for example, only one in ten males can reproduce and reproduction depends on hunting performance or success in raids, it would be more rational to engage in high-risk actions than in situations that limit the differences between the reproductive success of males within a population. Please keep in mind that the pace of the development depends on the non-random asymmetry in reproductive success, also known as *selective pressure*. The prediction would be that social structures allowing for greater selective pressure would be selected on the level of the group for the two aforementioned reasons: faster adaptation and, even more importantly, greater motivation to take risks during hunting and intergroup conflicts.

While the female choice of nice guys and good fathers—though possibly the most destructive force affecting the proliferation of the group—is relatively uninteresting, because it just slows down the process, the bias of the medium is highly instructive. We shall see that the nature of the medium will guide the development of the system, which will then shape its elements, including the cognitive faculties of our ancestors.

#### The Bias of the Medium

We will now discuss the media channel between hunting or combat behavior and reproduction. We are mainly interested in three issues: (1) the development of the backchannel and its media, (2) the type of behavior it results in and (3) the effect on the evolution of the organisms involved. We shall see that the behavior adapts on different timescales: on the level of genes, on the level of culture and learning and on the level of instantaneous adaptation to the situation. We will also see that the logic of the channel influences the nature of conflicts as well. To illustrate this principle, we will discuss two media types previously mentioned: trophy display and storytelling.

If this transmission of information is fulfilled by trophies, conflict between groups becomes primarily about obtaining trophies. The best approach for males would then be to find strategies to gain the desired trophies, while putting themselves in the least jeopardy possible. One good strategy might be working in a team with comrades-in-arms to kill isolated weak individuals one after another in a raid or an ambush attack.

However, when stories circulate, the logic of the intergroup conflicts will alter significantly, because the use of language allows combat behavior to be depicted with more precision. To pick the strongest opponent—as heroes do in Greek mythology—or to brave a superior number of enemies can make appealing stories. A significant bias of the medium of storytelling is *unnecessary heroism*, based on the fact that one great heroic deed makes a better story than continuous reliability—which might be more important for victories than single acts that overcame great risks. This entails that early wars were perhaps shaped more by strategies for individuals to shine as heroes than to win the battle.

An important difference between natural signs (such as trophy display) and storytelling is how the reliability of the content can be ensured. While storytelling can only work as a reliable medium when the content of the story is confirmed by other witnesses or verified by other evidence, trophies and other forms of natural signs, in general, do not need any validation.

In the previous paragraphs I tried to exemplify the priority of the medium and how the media of the backchannel shape not only male behavior, but also the development of the evolutionary system. In the next section we will discuss the dimensions of uncertainty.

### Dimensions of Uncertainty

**FIGURE 2:**
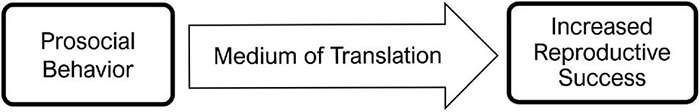
*The backchannel*: The backchannel model can explain both prosocial behavior (altruism) and the development of a medium of translating past action into reproductive success. This translation has two aspects: the transformation of knowledge of past actions into an image (see Figure 5) and the development of the technical part of the backchannel: the transmission of the information about past actions over time and space (from display of natural signs to linguistic communication; see Figure 4).

The toy model had the function of exemplifying a very general process that could be realized in many different ways, rendering the elements that fulfill the function in the model only examples. Prosocial behavior shall be translated into superior reproduction: this is the general form of the *attractor* (Figure 2):

The model of the process includes three variables:

(α)Increased Reproductive Success
(β)Medium of Translation
(γ)Prosocial Behavior

I shall discuss all three variables briefly.

#### (α) Female Choice and Its Functional Equivalents

In the toy model, the audience for trophy-displaying behavior or storytelling seems to be a single female choosing a mate based on the representation of his past actions. There are certainly other possibilities to fulfill the function required by the model. For instance, it would be possible that the relation between trophy display or storytelling and female choice is indirect: trophy display or storytelling could influence the social status of males and the female chooses a male with high status. They could also both be combined. For instance, trophy display or storytelling could have a broader audience that includes females.

**FIGURE 3:**
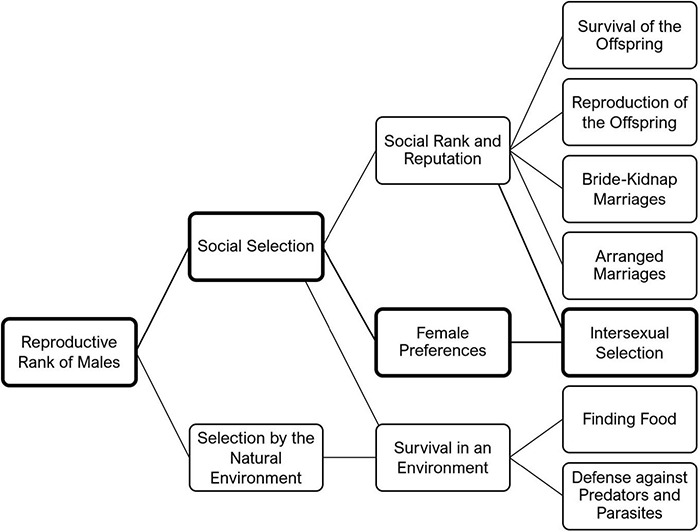
Reproductive rank of males in small-scale societies: The figure shows that there are many factors contributing to the reproductive fitness of males, and that the force on which the backchannel model is built (intersexual selection) is only one force among many others.

In any case, the communicated past actions change the future treatment of the individual whose past actions are conveyed. The channel broadens with cognitive and cultural development. An important step is made when individuals gain the ability to transform knowledge of an individual’s actions into respect or contempt toward the individual in question or even construct a consistent image of the individual. The general claim can be reduced to the idea that transmissions of individuals’ past actions influence their reproductive rank. Figure 3 shows many different ways this can happen, one having already been mentioned: that the presentation of a past action alters the doer’s social status and females choose males with high status. There also might be a correlation between combat performance and bride-kidnap marriages, as well as between reputation and arranged marriages. Social rank can also influence the survival of offspring. It has been shown, for instance, that children of good hunters have a better chance of survival. They are more sheltered and cuddled, protected from danger, and if a child of a good hunter is seriously ill, the tribe is less likely to relocate (Smith, 2004).

In stratified and modern societies, the process is more complex, since in stratified societies people are sometimes born into classes or castes and in modern capitalistic societies resources and property play a major role and can influence female mate choice (Buss, 1994), arranged marriages, the survival of the offspring, and their reputation (Hawkes, 2001).

Reputation built on circulated stories can also influence reciprocal relations in at least two ways: first, it can secure them, because anyone with the reputation of being a cheater can be excluded from co-operation (Nowak and Sigmund, 2005; Sperber and Baumard, 2012). Second, in egalitarian societies (but not necessarily in societies in which resources are unequally distributed) high-ranking individuals often receive more than those with a lower rank in reciprocal exchanges (Barclay, 2013).

However, the most remarkable point seems to be that in human societies the offspring inherit some of their family’s reputation. This makes fatal forms of altruism, such as *extreme self-sacrifice*, explainable in terms of inclusive fitness. It can be an evolutionarily stable strategy to fulfill a suicide mission—if this fosters the reproduction of one’s siblings and cousins (Blackwell, 2008) or one’s offspring. That entire families can be subject to reputation (just as individuals can) should be considered an adaptation to the attractor of the system. In other words, a society in which reputation can be attributed to families will, *ceteris paribus*, displace societies in which this is not the case.

In the last few paragraphs, we examined some dimensions of uncertainty concerning the realization of reproductive success. We found many functional equivalents to direct intersexual selection. This viability of the function’s realization not only increases the speed of the process but also makes it more robust.

#### (β) Storytelling in a Broad Sense

**FIGURE 4:**
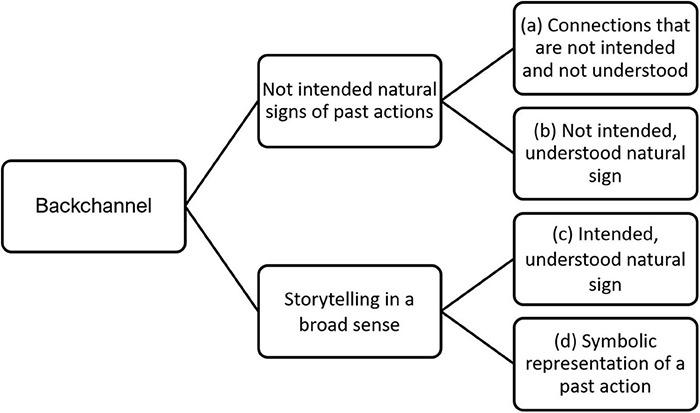
Media of the backchannel: The four stages toward language use.

Though many different types of media can fulfill the function of the backchannel, they can generally be categorized into four classes: (a) connections that are not intended and not understood, (b) not intended, understood natural signs, (c) intended, understood natural signs and (d) symbolic representation (see Figure 4). (b)-(d) we call *representational* since the audience can picture the past action.

**FIGURE 5:**
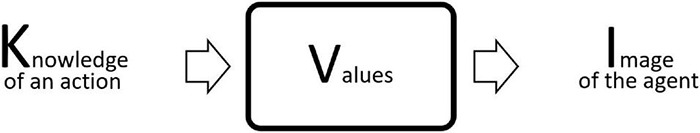
Values as a black box: The model explains altruistic actions. The knowledge of an agent’s action is transformed into an image of the agent according to the values of a society. Such altruistic actions are evolutionarily stable if the costs of the altruistic actions are lower than the benefits of the signal that shapes the image of the agent.

*Storytelling in a broad sense* can be performed by many means. It is defined here as intentionally transmitting information about a non-present action. This includes traditional storytelling via language but also the use of other technical media such as natural signs; for example, trophy display. In both cases, the audience imagines an absent event or action. From this perspective language is an *instruction of imagination* (Dor, 2015).

However, the function-first approach to the evolution of a trait (von Heiseler, 2020) would suggest that the forerunner of a representational account would have been a structure in which information about a non-present action is somehow objectively transmitted, creating a reproductive advantage despite not being understood by sender and receiver. For example, females could prefer males displaying hunting trophies without understanding what the trophies represent. Therefore, developing a drive in males to present trophies could be beneficial—even without the intention to convey the action that is implied by the trophy. This would be a backchannel without being representational.

Putting together the four media shown in Figure 4 into an evolutionary narrative, three explanatory gaps would need to be overcome: (Gap 1) (a) Connections that are not intended and not understood to (b) not intended understood natural signs. (Gap 2) (b) From not intended understood natural signs to (c) intended natural signs. (Gap 3) (c) From intended natural signs to (d) simple sentences.

(Gap 1) If female choice can improve by gaining understanding of the natural sign (e.g., how a hunting trophy implies a successful past action by the presenter), it might develop because it would benefit the reproduction of the genes of the choosing female.
(Gap 2) The presentation of evidence for a past action will be probably much more effective and flexible if presenters have the intention to communicate their past action.
(Gap 3) It has been shown (von Heiseler, 2020) that the display of hunting or war trophies can imply a conceptual structure similar to that of syntax in sentences: the trophy symbolizes the patient, the displayer the agent, and the verb—to kill—is implied by the condition of the trophy. Therefore, a gradual evolutionary narrative can be told from a trophy presentation to a simple signed sentence in which the previously implied verb is expressed as a mimetic gesture (von Heiseler, 2019). The direction of the gesture signifying the verb marks the semantic roles. For instance, if the verb is ‘to kill with a stone’, the gesture is directed from the agent to the patient— or, in our case, from the displayer to the trophy, while the opposite direction would switch the semantic roles (which would not make any sense, since the displayer is still alive).

#### (γ) Prosocial Behavior

We defined prosocial behavior in terms of the proliferation of the group and interpreted it rather narrowly in terms of food sharing and defending and expanding the territory. However, the proliferation of a group depends on many factors, such as overcoming natural boundaries (for example, oceans, ice, mountains, and deserts), hygiene and healthcare and, maybe most importantly, improving group coherence in a way that allows greater and better organized social structures—which then may result in new lifestyles and in the acceleration of cultural and technological evolution (Henrich, 2017).

The historical development of prosocial behavior is connected to moral values. We defined moral values as the transformation rule of how the actions of an agent are translated into the image of an agent. In the psychological dimension, a value is a black box in an individual’s head. The input is a belief about a person’s actions and the output is an image of the person. It is remarkable how this complex process takes place without any effort, even when we consume a piece of fiction.

### The Explanatory Power of the Hypothesis Presented in This Paper

We shall see that the model can not only explain cooperation and the evolution of language, but also theory of mind, episodic memory, and the development of a moral system based on the cognitive ability to translate the knowledge of others’ actions into a consistent image of the individual in question. We shall also see that the model also provides us with a suggestion of an evolutionary function of the *self* as an organ of impression management, as suggested by Barkow (1989).

The hypothesis presented in this paper predicts that many distinctive human cognitive capacities are selected (1) for storytelling, (2) for actions aiming for reputation, recognition, and respect, including self-respect, and (3) for social cognition to find the behavior with the lowest costs and the greatest reputational benefits:

(1) Many distinctive human cognitive capacities can be explained as adaptations for storytelling: the ability to represent and comprehend non-present actions, including understanding of the attention of others, the comprehension of mimetic signs and syntactical structures (which is necessary when conferring non-present actions), episodic memory and social cognition—for instance, the ability to figure out when to begin which story and adapt the story to the present audience. It could also be argued that understanding the beliefs of others (theory of mind) could have evolved in the context of storytelling, since successful storytelling is only possible when storytellers keep two things in mind: what they have already told—meaning the perspective of the audience—and the whole story. This point also includes conventions and techniques essential for demonstrating storytelling ability in a given society.
(2) Non-human animals adapt to interaction with the environment. In contrast, humans *compete* in how they interact with the environment (Ridley, 1993). This also changes the economics of the group fundamentally: resources are shared freely between all members of the group in most small-scale societies (Gurven and Hill, 2009), since sharing can be translated into reputation, which is relevant for reproductive success. The adaptation to actions that are done for reputation varies historically. This makes them a moving target, putting the ability to learn from others under selective pressure—as demonstrated by the high plasticity of the human brain. This mindset is also the foundation of the concept of work and functional differentiation and plays a major role in the production of surplus.
(3) The knowledge of values is essential for identifying behavior that will improve one’s image at minimal cost (while ignorance of the values of a society might incur high costs with little or no benefit). This led to the evolution of sensitivity to the prevalent values of a society and moral conformism. Since individuals often need to act instantly, the social information has to be present all the time. Only then can adjustments be made according to new information in time-critical situations. This would predict that any neurotypical individual would have at least three types of information *always present*: (a) information about values (how knowledge about actions is translated into respect and contempt), (b) information about the status and relations of others, and (c) information about one’s own status and relations to others. This would suggest: (I) Deep identification with the values of the society we live in. Values must be internalized, because only then can instinctive and emotional reactions be led by them. (II) An innate drive to be interested in gossip: that we are fascinated by stories about people we know, especially if their behavior includes extreme forms of altruism or moral transgressions. (III) That we developed a system to evaluate our own status, which needs to be active all the time (for instance, based on serotonin) and that, on the path toward *behavioral modernity*, a new basic instinct, *the desire for recognition* (cf. Hegel, 1807/1980; Kojève, 1975) evolved, which can also be seen as an essential need for reputation (cf. Smith, 1759).

Our model also explains how the norms of a society are connected to our identity—to what we essentially believe we are. This is why Cassio in Shakespeare’s *Othello* cries out: “Reputation, reputation, reputation! Oh, I have lost my reputation! I have lost the immortal part of myself, and what remains is bestial.”

## Conclusion

In this essay I have tried to explain many aspects of human evolution in terms of equilibrium selection: humans evolved by a process of adaptation to the equilibrium of their group by adjusting to the best individual reproductive strategy and, in a second step, a selection between different equilibria taking place. I claimed that this evolutionary system would have naturally developed a backchannel between the two levels, making it possible for the selection of the lower level to be informed by the selection of the higher level, as the individual contribution to the proliferation of the group is translated into reproduction. For implementing this backchannel, language and a sensitivity to values are necessary. Altruistic behavior is evolutionarily stable when the costs of the altruistic act are lower than the benefit of signaling it.

Due to the limited space, I could only present a first sketch of the hypothesis. It is my conviction that further adaptation of the toy model to reality should be a collective enterprise for two reasons: First, I can only see what I can see and will have a bias toward the mechanisms I described, while the further discussion would need to include positions that are more critical of the presented ideas that I can be. Second, adaptation to the real world means taking into account empirical data, concepts, and theories from many different fields, such as linguistics, evolutionary biology, paleoanthropology, neuroscience, primatology, anthropology, ethology, sociology, and psychology. For these and other reasons, the analysis of the developments presented here could only be preliminary. An important test of the hypothesis would be to investigate whether it produces a consistent evolutionary narrative, which is beyond the scope of this paper.

## Author Contributions

The author confirms being the sole contributor of this work and has approved it for publication.

## Conflict of Interest

The author declares that the research was conducted in the absence of any commercial or financial relationships that could be construed as a potential conflict of interest.

## Publisher’s Note

All claims expressed in this article are solely those of the authors and do not necessarily represent those of their affiliated organizations, or those of the publisher, the editors and the reviewers. Any product that may be evaluated in this article, or claim that may be made by its manufacturer, is not guaranteed or endorsed by the publisher.

## Footnotes

1 Though in the literature collaboration and altruism are sometimes used more or less synonymously, it seems useful to point out how the concepts relate to each other. Collaboration is defined as a relation between at least two organisms whereby all partners benefit in the long run. The question emerges: what are the mechanisms that stabilize such collaborations and control cheating and free riding? For formulating hypotheses about such stabilization mechanisms, the concept of altruism is useful. Altruism is here defined as a costly behavior that benefits others or the group. Altruistic behavior expresses itself in *not cheating* in reciprocal relations, in *not free riding* in common-good games, and sometimes even in laying down one’s life for the group. To explain altruism in Darwinian terms is to explicate the benefits of altruistic behavior, which need to be higher than its costs. This makes the term incompatible with folk psychological concepts of altruism. When we solve the problem of altruism (by explaining why people do not cheat or do not free ride) we can solve the problem of cooperation.

2 I use *ancestors* in this paper in a rather loose way, because technically only a fraction of early *Hominini* are our direct ancestors. The precise wording would be *our ancestors and their relatives (whose lines became extinct)*.

3 The group including modern humans and all extinguished taxa more closely related to modern humans than any other living taxon (Wood, 2011).

4 The key to fast-developing cumulative culture is *overimitation*. While children imitate others (such as their parents, older siblings, peers, the majority, the successful, the prestigious) even if they do not see any instrumental purposes (Hoehl et al., 2019), non-human primates do not over-imitate (Horner and Whiten, 2005). In other words, non-human primates do not imitate any behavior when they do not see any instrumental purpose, but rather optimize the observed behavior for efficiency. Only when overimitation is in place does cultural development produce clear cultural difference even between people living in the same environment.

5 There might be some ideological concerns arising in the mind of the reader. Nevertheless, we should be careful about projecting our political agendas onto our past. The idea that we need to justify our position from nature is a variation of the *naturalistic fallacy*. In other words, even if it could be proven that our female ancestors never hunted nor engaged in intergroup conflicts (which would certainly be false) this would not lead to the conclusion that women today should not freely choose any occupation they like.

6 This only works because high-quality individuals can produce the same signal with lower costs compared to their resources (Grafen, 1990). As a consequence, any model in which all agents are constructed equally cannot predict much about the evolution of costly signaling.

## References

[B1] Alexander R. D. (2008). Evolution and human society. Human Behavior and Evolution Society. *Newsletter.* 5–15.

[B2] Annan J. Blattman C. Mazurana D. Carlson K. (2011). Civil war, reintegration, and gender in Northern Uganda. *J. Confl. Resolut.* 55 877–908. 34479000

[B3] Ardrey R. (1976). *The Hunting Hypothesis: A Personal Conclusion Concerning the Evolutionary Nature of Man.* New York, NY: Atheneum.

[B4] Barclay P. (2013). Strategies for cooperation in biological markets, especially for humans. *Evol. Hum. Behav.* 34 164–175. 10.1016/j.evolhumbehav.2013.02.002

[B5] Barkow J. H. (1989). *Darwin, Sex, and Status: Biological Approaches to Mind and Culture.* Toronto, Ont: University of Toronto Press. 10.3138/9781442673724

[B6] Bickerton D. (2007). Language evolution: a brief guide for linguists. *Lingua* 117 510–526. 10.1016/j.lingua.2005.02.006

[B7] Bickerton D. Szathmáry E. (2011). Confrontational scavenging as a possible source for language and cooperation. *BMC Evol. Biol.* 11:261. 10.1186/1471-2148-11-261 21933413 PMC3188516

[B8] Blackwell A. (2008). *Middle-Class Martyrs: Modeling the Inclusive Fitness Outcomes of Palestinian Suicide Attack.* Eugene, OR: University of Oregon, Anthropology Department.

[B9] Bowles S. Gintis H. (2003). “The origins of human cooperation,” in *Genetic and Cultural Evolution of Cooperation*, ed. Hammerstein P. (Cambridge MA: MIT Press), 429–444.

[B10] Boyd R. Richerson P. J. (1990). Group selection among alternative evolutionarily stable strategies. *J. Theor. Biol.* 145 331–342. 10.1016/s0022-5193(05)80113-4 2232821

[B11] Boyd R. Richerson P. J. (2005). “Solving the puzzle of human cooperation,” in *Evolution and Culture*, eds Levinson S. Jaisson P. (Cambridge MA: MIT Press), 105–132.

[B12] Boyd R. Richerson P. J. (2009). Culture and the evolution of human cooperation. *Philos. Trans. R. Soc. Biol. Sci.* 364 3281–3288. 10.1098/rstb.2009.0134 19805434 PMC2781880

[B13] Britten R. J. (2010). Transposable element insertions have strongly affected human evolution. *Biol. Sci. Evol.* 107 19945–19948. 10.1073/pnas.1014330107 21041622 PMC2993358

[B14] Burbano H. A. Green R. E. Pääbo S. (2012). Analysis of human accelerated DNA regions using archaic hominin genomes. *PLoS One* 7:e32877. 10.1371/journal.pone.0032877 22412940 PMC3296746

[B15] Bush E. C. Lahn B. (2008). A genome-wide screen for noncoding elements important in primate evolution. *BMC Evol. Biol.* 8:17. 10.1186/1471-2148-8-17 18215302 PMC2242780

[B16] Buss D. (1994). *The Evolution of Desire.* New York, NY: Basic Books.

[B17] Chagnon N. A. (1968). *Yanomamö: The Fierce People.* Boston, MA: Holt McDougal.

[B18] Chomsky N. (2006). *Language and Mind.* Cambridge: Cambridge University Press. 10.1017/CBO9780511791222

[B19] Coppens I. (2004). Geotektonik, klima und der ursprung des menschen. *Spektrum Wiss. Dossier Evol. Menschen* 4 6–13.

[B20] Darwin C. (1859). *On the Origin of Species by Means of Natural Selection, or the Preservation of Favoured Races in the Struggle for Life.* London: John Murray. 10.5962/bhl.title.162283 PMC5184128 30164232

[B21] David-Barrett T. Dunbar R. (2016). Language as a coordination tool evolves slowly. *R. Soc. Open Sci.* 3:160259. 10.1098/rsos.160259 28083091 PMC5210673

[B22] Deacon T. W. (1997). *The Symbolic Species: The Co-Evolution of Language and the Brain.* London: W.W. Norton & Co.

[B23] Dor D. (2015). *The Instruction of Imagination – Language as a Social Communication Technology.* Oxford: Oxford University Press. 10.1093/acprof:oso/9780190256623.001.0001

[B24] Fehr E. Fischbacher U. Gächter S. (2002). Strong reciprocity, human cooperation and the enforcement of social norms. *Hum. Nat.* 13 1–25. 10.1007/s12110-002-1012-7 26192593

[B25] Fisher R. A. (1930). *The Genetical Theory of Natural Selection.* Oxford: Clarendon. 10.5962/bhl.title.27468

[B26] Fitch T. W. (2010). *The Evolution of Language.* Cambridge: Cambridge University Press. 10.1017/CBO9780511817779

[B27] Gagneux P. Boesch C. Woodruff D. S. (1999). Female reproductive strategies, paternity and community structure in wild West African chimpanzees. *Anim. Behav.* 57 19–32. 10.1006/anbe.1998.0972 10053068

[B28] Giardini F. Conte R. (2012). Gossip for social control in natural and artificial societies. *Simulation* 88 18–32. 10.1177/0037549711406912

[B29] Goodall J. (1986). *The Chimpanzees of Gombe: Patterns of Behavior.* Cambridge MA: Harvard UP.

[B30] Grafen A. (1990). Biological signals as handicaps. *J. Theor. Biol.* 144 517–546. 10.1016/s0022-5193(05)80088-8 2402153

[B31] Gurven M. Hill K. (2009). Why do men hunt? A re-evaluation of “Man the Hunter” and the sexual division of labor. *Curr. Anthropol.* 50 51–62. 10.1086/595620 19579355

[B32] Hamilton W. D. (1964). The genetical evolution of social behaviour I & II. *J. Theor. Biol.* 7, 1–52. 10.1016/0022-5193(64)90039-6 5875341

[B33] Hawkes K. (2001). “Is meat the Hunter’s property? Big game, ownership, and of hunting and sharing,” in *Meat-Eating and Human Evolution*, eds Stanford C. B. Ciochon R. Wood B. (Oxford: Oxford Univ Press).

[B34] Hegel G. W. (1807/1980). *Phänomenologie des Geistes (Kritische Edition, Gesammelte Werke)*, 9 Edn. Hamburg: Rheinisch-Westfälische Akademie der Wissenschaften. 10.28937/978-3-7873-3389-9

[B35] Henrich J. (2004). Cultural group selection, coevolutionary processes and large-scale cooperation. *J. Econ. Behav. Organ.* 53 3–35. 10.1017/sjp.2016.101 28065201

[B36] Henrich J. (2017). *The Secret of Our Success: How Culture is Driving Human Evolution, Domesticating Our Species, and Making Us Smarter.* Princeton, NJ: Princeton University Press. 10.1515/9781400873296

[B37] Henrich J. Boyd R. (2001). Why people punish defectors. *J. Theor. Biol.* 208 79–89. 10.1006/jtbi.2000.2202 11162054

[B38] Hoehl S. Keupp S. Schleihauf H. McGuigan N. Buttelmann D. Whiten A. (2019). Over-imitation: a review and appraisal of a decade of research. *Dev. Rev.* 51 90–108. 10.1016/j.dr.2018.12.002

[B39] Horner V. Whiten A. (2005). Causal knowledge and imitation/emulation switching in chimpanzees (*Pan troglodytes*). *Anim. Cogn.* 8 164–181. 10.1007/s10071-004-0239-6 15549502

[B40] Hughes J. F. Skaletsky H. Pyntikova T. Graves T. A. Saskia K. M. van Daalen P. J. (2010). Chimpanzee and human Y chromosomes are remarkably divergent in structure gene content. *Nature* 463 536–539. 10.1038/nature08700 20072128 PMC3653425

[B41] Humphrey N. (1976). “The social function of intellect,” in *Growing Points in Ethology*, eds Bateson P. Hinde R. (Cambridge: Cambridge University Press), 303–317.

[B42] Hunt K. D. McGrew W. C. (2002). “Chimpanzees in the dry habitats at Assirik, Senegal, and at Semliki Wildlife Reserve, Uganda,” in *Behavioural Diversity in Chimpanzees and Bonobos*, eds Boesch C. Hohmann G. Marchant L. F. (Cambridge: Cambridge University). 10.1017/CBO9780511606397.005

[B43] Jiang Y. Bolnick D. I. Kirkpatrick M. (2013). Assortative mating in animals. *Am. Nat.* 181 E125–E138. 10.1086/670160 23669548

[B44] Jones J. M. Henshaw A. G. (2019). Fisher’s lost model of runaway sexual selection. *Evolution* 74 487–494. 10.1111/evo.13910 31886520

[B45] Kojève A. (1975). *Hegel.* Frankfurt am Main: Suhrkamp.

[B46] Lahn B. Dorus S. Vallender E. J. Evans P. D. Anderson J. R. Gilbert S. L. (2004). Accelerated evolution of nervous system genes in the origin of *Homo sapiens*. *Cell* 119 1027–1040. 10.1016/j.cell.2004.11.040 15620360

[B47] Lande R. Kirkpatrick M. (1988). Ecological speciation by sexual selection. *J. Theor. Biol.* 133 85–98. 10.1016/S0022-5193(88)80026-2 3226143

[B48] Maynard Smith J. Szathmáry E. (1995). *The Major Transitions in Evolution.* New York, NY: Oxford University Press.

[B49] Maynard Smith J. Szathmáry E. (2000). *The Origins of Life: From the Birth of Life to the Origin of Language.* Oxford: Oxford University Press.

[B50] Mayr E. (1942). *Systematics and the Origin of Species from the Viewpoint of a Zoologist.* New York, NY: Columbia University Press.

[B51] Mayr E. (1982). *The Growth of Biological Thought: Diversity, Evolution, and Inheritance.* Harvard, MA: Harvard University Press.

[B52] Miller G. (2000). *The Mating Mind: How Sexual Choice Shaped the Evolution of Human Nature.* London: Heineman.

[B53] Miller G. (2007). Sexual selection for moral virtues. *Q. Rev. Biol.* 82 97–125. 10.1086/517857 17583267

[B54] Mitani J. C. Watts D. P. Amsler S. J. (2010). Lethal intergroup aggression leads to territorial expansion in wild chimpanzees. *Curr. Biol.* 20 R507–R508. 10.1016/j.cub.2010.04.021 20620900

[B55] Nakamaru M. Kawata M. (2004). Evolution of rumours that discriminate lying defectors. *Evol. Ecol. Res.* 6 261–283.

[B56] Nishida T. Hiraiwa-Hasegawa M. (1986). “Chimpanzees and bonobos: cooperative relationships among males,” in *Primate Societies*, eds Smuts B. Cheney D. Seyfarth R. Wrangham R. Struhsaker T. (Chicago, IL: The University of Chicago Press), 165–177.

[B57] Nowak M. Sigmund K. (2005). Evolution of indirect reciprocity. *Nature* 437 1291–1298. 10.1038/nature04131 16251955

[B58] Penn D. J. Szamado S. (2020). The handicap principle: how an erroneous hypothesis became a scientific principle. *Biol. Rev.* 95 267–290. 10.1111/brv.12563 31642592 PMC7004190

[B59] Pepper J. W. Mitani J. C. Watts D. P. (1999). General gregariousness and specific social preferences among wild chimpanzees. *Int. J. Primatol.* 20 613–632. 10.1023/A:1020760616641

[B60] Pollard K. Salama S. Lambert N. Lambo T. M. (2006). An RNA gene expressed during cortical development evolved rapidly in humans. *Nature* 443 167–172. 10.1038/nature05113 16915236

[B61] Prabhakar S. Noonan J. Paabo S. Rubin E. (2006). Accelerated evolution of conserved noncoding sequences in humans. *Science* 314:786. 10.1126/science.1130738 17082449

[B62] Richerson P. Boyd R. (2005). *Not by Genes Alone: How Culture Transformed Human Evolution.* Chicago, IL: University of Chicago Press. 10.7208/chicago/9780226712130.001.0001

[B63] Ridley M. (1993). *The Red Queen: Sex and the Evolution of Human Nature Paperback.* New York, NY: Viking Press.

[B64] Smith A. (1759). *Theory of Moral Sentiments.* Edinburgh: A. Millar; A. Kincaid; J. Bell.

[B65] Smith E. A. (2004). Why do good hunters have higher reproductive success? *Hum. Nat.* 15 343–364. 10.1007/s12110-004-1013-9 26189411

[B66] Sperber D. Baumard N. (2012). Moral reputation: an evolutionary and cognitive perspective. *Mind Lang.* 27 495–518. 10.1111/mila.12000

[B67] Sterelny K. (1997). *The Evolution of Agency and Other Essays.* Cambridge: Cambridge University Press.

[B68] Stumpf R. M. Boesch C. (2006). The efficacy of female choice in chimpanzees of the Taï Forest, Côte d’Ivoire. *Behav. Ecol. Sociobiol.* 60 749–765. 10.1007/s00265-006-0219-8

[B69] Tomasello M. (2003). “On the different origins of symbols and grammar,” in *Language Evolution*, eds Christiansen M. H. Kirby S. (Oxford: Oxford Univ. Press), 94–110. 10.1016/j.biosystems.2019.04.011

[B70] Tooby J. Cosmides L. (1992). “The psychological foundations of culture,” in *The Adapted Mind: Evolutionary Psychology and the Generation of Culture*, eds Barkow J. H. Cosmides L. Tooby J. (Oxford: Oxford University Press), 19–136.

[B71] Tooby J. DeVore I. (1987). “The reconstruction of hominid evolution through strategic modeling,” in *The Evolution of Human Behavior: Primate Models*, eds Kinzey W. G. Suny Series in Primatology (New York, NY: State University of New York).

[B72] Trivers R. (1972). “Parental investment and sexual selection,” in *Sexual Selection and the Descent of Man*, ed. Campbell B. (Chicago, IL: Aldine), 136–179. 10.4324/9781315129266-7

[B73] Tucker M. Miller G. (2015). *Mate: Become the Man Women Want.* Boston, MA: Little, Brown and Company.

[B74] von Heiseler T. N. (2019). Syntax of testimony: indexical objects, syntax, and language or how to tell a story without words. *Front. Psychol.* 10:477. 10.3389/fpsyg.2019.00477 30967805 PMC6438894

[B75] von Heiseler T. N. (2020). The social origin of the concept of truth – how statements are built on disagreements. *Front. Psychol.* 11:733. 10.3389/fpsyg.2020.00733 32411047 PMC7198879

[B76] Watts D. Mitani J. (2001). Boundary patrols and intergroup encounters in wild chimpanzees. *Behaviour* 138 299–327. 10.1007/BF02629601 12426461

[B77] Weatherhead P. J. Robertson R. J. (1979). Offspring quality and the polygyny threshold: ‘the sexy son hypothesis’. *Am. Nat.* 113 201–208. 10.1086/283379

[B78] Whiten A. Byrne R. (1988). Tactical deception in primates. *Behav. Brain Sci.* 11 233–273. 10.1017/s0140525x00049682

[B79] Williams G. (1966). *Adaptation and Natural Selection.* Princeton, NJ: Princeton University Press.

[B80] Wood B. (2011). *Wiley-Blackwell Encyclopedia of Human Evolution.* Hoboken, NJ: Wiley-Blackwell. 10.1002/9781444342499

[B81] Wrangham R. W. Glowacki L. (2012). Intergroup aggression in chimpanzees and war in nomadic hunter-gatherers. *Hum. Nat.* 23 5–29. 10.1007/s12110-012-9132-1 22388773

[B82] Zahavi A. (1975). Mate selection – a selection for a handicap. *J. Theor. Biol.* 53 205–214. 10.1016/0022-5193(75)90111-3 1195756

[B83] Zahavi A. (1993). The fallacy of conventional signalling. *Philos. Trans. R. Soc. Lond. B* 340 227–230. 10.1098/rstb.1993.0061 8101657

[B84] Zlatev J. (2014). “The co-evolution of human intersubjectivity, morality and language,” in *The Social Origins of Language*, eds Dor D. Knight C. Lewis D. (Oxford: Oxford University Press), 249–266. 10.1093/acprof:oso/9780199665327.003.0018

